# 非小细胞肺癌DNA甲基化研究进展

**DOI:** 10.3779/j.issn.1009-3419.2010.08.14

**Published:** 2010-08-20

**Affiliations:** 210002 南京，南京大学医学院临床学院（南京军区南京总医院）肿瘤内科 Department of Medical Oncology, Jinling Hospital, Medical School of Nanjing University, Nanjing 210002, China

**Keywords:** 肺肿瘤, 甲基化, 抑癌基因, 分子标记, Lung neoplasms, Methylation, Tumor suppressor gene, Biomarker

## Abstract

基因组DNA甲基化是目前发现的最主要的一种表观遗传修饰形式，高甲基化（hypermethylation）的DNA染色质构象发生改变，导致抑癌基因转录失活，在肿瘤发生发展中具有重要意义。近年来，DNA甲基化在肺癌，主要是非小细胞肺癌（non-small cell lung cancer, NSCLC）的研究中取得较大进展，为NSCLC早期诊断、风险评估、预后判断和干预治疗提供了新的靶点。

## Abstract (en)

Genomic DNA methylation is a major form of epigenetic modification. Hypermethylation could affect the binding of transcription factors to DNA and change the structure of chromatin resulting in silence of tumor suppressor genes, which plays an important role in cancer initiation and progression. In recent years, the study of DNA methylation in lung cancer, mostly in non-small cell lung cancer, has made great progress and become a new target for early detection, risk assessment, prognosis and cancer therapy.

肺癌是世界范围内发病率与死亡率均位于第一位的肿瘤，其中非小细胞肺癌（non-small cell lung cancer, NSCLC）占据85%-90%。肺癌的发生是一个多步骤，多因素参与的复杂的生物学过程，涉及遗传学（genetic）与表观遗传学（epigenetic）信息的改变。DNA甲基化是最重要的一种表观遗传修饰方式，高甲基化所致基因转录失活，与胚胎发育和肿瘤疾病有着密切关系^[1]^。在本世纪初，仅有为数不多的单个抑癌基因（tumor suppressor gene, TSG）甲基化在小规模NSCLC样本中的研究，近十年来，越来越多的基因在肺癌中的甲基化状态被报道，并且，甲基化的DNA片段也可以在外周血和支气管上皮脱落细胞中检测到，某些基因甲基化还与患者不良预后或肿瘤复发有关，新的高通量高灵敏度的甲基化检测方法也得到了较快发展。本文对这方面的进展予以综述。

## 1 DNA甲基化的概念

DNA甲基化，指DNA甲基转移酶（DNA methyltransferase, DNMT）催化S-腺苷甲硫氨酸作为甲基供体，将CpG二核苷酸5'端的胞嘧啶转变为5'甲基胞嘧啶的过程。这种DNA修饰方式并没有改变基因序列，但能引起染色质结构、DNA稳定性及DNA与蛋白质相互作用方式的改变，从而控制基因表达^[1]^。肿瘤组织中DNA常见重复序列、组织特异性基因、印记基因的相关CpG位点往往呈去甲基化状态，因此整体甲基化水平比正常组织低20%-60%；但局部区域如抑癌基因启动子CpG岛却发生异常高甲基化，使染色质螺旋程度增加，基因转录受到抑制^[2]^。近来的证据^[3]^表明，DNA甲基化和组蛋白的化学修饰，如乙酰化、甲基化、泛素化（ubiquitination）、磷酸化、ADP核糖基化（ADPribosylation）等存在相互作用，共同调节基因转录。一些研究^[4]^还表明DNA甲基化直接影响组蛋白的乙酰化和甲基化状态。

DNA甲基化是一个可逆的过程，高甲基化沉默的基因可以被DNMTs抑制剂如5-氮杂胞苷（5-aza-2′-deoxycytidine, 5-Aza-dC）恢复表达，而组蛋白去乙酰化酶（hi stone deacetylase, HDACs）抑制剂如trichostatin A（TSA）等可显著增加DNMTs抑制剂的激活效应^[5]^。

## 2 NSCLC抑癌基因甲基化概述

最初，NSCLC中甲基化的研究集中于单个或少数功能相对明确的抑癌基因，这些研究的内容包括观察甲基化在肿瘤组织和正常组织的差异，以及与临床病理参数的联系，进而作为预测或预后指标。如，王锐等^[6]^发现，*Fibulin3*基因在NSCLC组织中的甲基化频率为43.1%（28/65），在相应正常组织仅为9.2%（6/65），*Fibulin3*高甲基化导致其mRNA和蛋白水平表达下调，并与肿瘤分化、分期和淋巴结转移有关；Yu等^[7]^报道，*EPHB6*甲基化引起的基因表达下调与远处转移风险的增加有关；而Choi等^[8]^报道，31.6%（31/98）的NSCLC组织检出*ADAMTS1*基因甲基化，与患者临床病理参数如年龄、性别、组织学类型和临床分期等无相关。通过这些研究，许多抑癌基因在NSCLC中的甲基化状态得到鉴定，并且证实DNA甲基化的临床应用价值，如早期检测、诊断、化学预防、治疗和预后。进一步的研究是联合检测多基因的甲基化状态，以明确NSCLC中甲基化频率较高的位点，作为潜在的生物标记。其中，有些基因得到了较广泛的关注，在多项独立研究中均被报道具有较高的甲基化频率，如*APC*、*CADM1*、*CDH1*、*CDH13*、*p14^ARF^*、*p16^INK4a^*、*DAPK*、*FHIT*、*GSTP1*、*MGMT*、*MLH1*和*RASSF1A*等^[9]^，但也有不一致的结果，解释有多种，如研究方法、人种以及肿瘤亚型的差异。

Toyooka等^[10]^研究发现，甲基化方式的差异不仅存在于NSCLC、小细胞肺癌（small cell lung cancer, SCLC）和类癌之间，在NSCLC的亚型间也存在，*APC*和*CDH13*甲基化在腺癌中显著高于鳞癌，而*p16*甲基化频率在鳞癌中高于腺癌。Gu等^[11]^分析NSCLC中9个基因（*p16*、*CDH1*、*TIMP3*、*RASSF1A*、*FHIT*、*APC*、*DAPK*、*MGMT*和*GSTP1*）的甲基化状态，发现腺癌的甲基化指数（methylation index, MI）显著高于鳞癌。DNA甲基化方式的差异可能也与某些基因型异常有关，如*EGFR*突变的腺癌中，*p16*和*CDH13*甲基化以及MI均低于野生型；相反，*K-ras*突变更常见于*p16*甲基化的组织，*K-ras*突变的MI也显著高于野生型^[12]^。推测甲基化参与EGFR和K-ras介导的肺腺癌发生过程，遗传学与表观遗传学异常相互作用，共同促进肿瘤发生。

随着分子生物学技术的发展，一些高通量的检测方法可以同时检测更多位点的甲基化。其中的一个方法是对亚硫酸氢盐处理的DNA进行半定量real time PCR（MethyLight），近来有3篇报道利用MethyLight方法研究NSCLC中27个位点^[13]^、腺癌中28个位点^[14]^和鳞癌中42个位点^[15]^的甲基化状态，结果均确定了一组敏感而特异的甲基化标记。Ehrich等^[16]^利用MALDI-TOF（matrix assisted laser desorption/ionization-time of flight）为基础的技术，分析96例患者的47个甲基化位点，发现*CLEC3B*、*MGP*、*RASSF1*、*SDK2*、*SERPINB5*和*XAGE1A*这6个基因的甲基化在癌组织中显著高于相应正常组织。另一项类似研究^[17]^分析288个肿瘤相关基因的启动子区域，发现28个潜在的标记性位点，其中5个在肺癌组织中进一步研究的结果确定*PAX3*和*PYCARD/ASC*是特异性的高甲基化位点。RLGS（restriction landmark genomic scanning）方法可以分析2 000个启动子序列，Dai等^[18]^对1 184个CpG岛的研究发现11个基因在肿瘤组织中有差别的甲基化，其中*GNAL*和*PDX1*基因的甲基化比例超过50%。Illumina Golden-Gate platform是一项新的高通量技术，Bibikova等^[19]^在腺癌中对807个基因的1 505个CpG位点进行分析，发现一组特异性的甲基化谱，其中的8个（ASCL2、CDH13、HOXA11、HOXA5、NPY、RUNX3、TERT和TP73）进一步经BSP测序法确认。

芯片技术的发展为DNA甲基化研究提供了更好的平台，使得甲基化研究不再针对某些特定位点，而能够在整个基因组水平上无偏倚地分析肿瘤与正常组织或细胞的差异，从而更深入地理解DNA甲基化在NSCLC中的发生方式，并提供新的标记物。通过基因表达芯片对细胞株以5-Aza-dC处理前后的分析，可以确定DNA甲基化的靶基因，Shames等^[20]^通过这种方法发现132个肿瘤特异性的甲基化位点，对其中45个进一步研究，确定7个（ALDH1A3、BNC1、CCNA1、CTSZ、LOX、MSX1和NRCAM）潜在的肺癌标记物。利用MIRA-assisted芯片（methylated CpG island recovery assay coupled with microarray analysis），Rauch等^[21]^从肺腺癌细胞A549和支气管上皮细胞NHBE中收集富含CpG区域的片段，与含有12 192个CpG岛的CpG芯片杂交，发现包括DLEC1、PAX7等基因在内的50个甲基化位点有显著差异，进一步的研究发现其中11个位点在鳞癌中的甲基化比例达到80%-100%^[22]^。

毫无疑问，这些高通量的检测方法可以迅速地鉴定许多潜在的甲基化标记物，但是采用不同方法报道的甲基化位点又各不相同，彼此之间很少有重复性，因此，这些新的标记物必须通过传统方法在原发肿瘤组织中进行验证，并进行优化组合，尽可能地排除人种、性别及肿瘤异质性等因素的影响。

## 3 游离DNA甲基化与NSCLC早期诊断及筛查

DNA甲基化是肿瘤发生中较频繁的早期事件，可作为NSCLC早期诊断和筛查的潜在标记。大量研究证实，甲基化的DNA片段也可以在外周血和支气管上皮脱落细胞中检测到，这种非侵袭性的检查方法使甲基化检测有了更好的应用前景。

外周血是比较理想的样本，肿瘤患者中存在高水平的循环DNA（circulating DNA），可能源自肿瘤细胞自分泌、裂解、坏死或凋亡等方式的释放，并往往与原发肿瘤有着相同的基因改变^[23]^。NSCLC组织中研究较多的一些甲基化位点几乎均在血浆/血清中得到了证实。如Hsu等^[24]^报道，63例肺癌血浆中，*BLU*、*CDH13*、*FHIT*、*p16*、*RARβ*和*RASSF1A*基因甲基化与相应肿瘤组织符合率依次为86%、87%、80%、75%、76%和84%，并且其中任意两者的甲基化对肿瘤诊断的敏感性和特异性可达73%和82%以上。本研究小组在NSCLC循环血DNA中筛选NSCLC敏感而特异的甲基化位点，先后发现*p16*、*DAPK*、*RASSF1A*、*SFRP1*和*KLK10*的甲基化比例分别为43.1%（28/65）、30.8%（20/65）、33.8%（27/80）、28.2%（22/78）和38.7%（30/78），与肺部良性疾病及正常对照者差异显著^[25-29]^，可作为NSCLC早期诊断的潜在标记。另一项研究^[30]^表明，NSCLC血浆中*p16*甲基化联合微卫星改变（microsatellite alterations）将诊断的敏感性提高到62%，*p16*甲基化与循环DNA含量的联合，可提高敏感性到80%，优于单独甲基化检测。因此，循环DNA甲基化与其它类型肿瘤标记物的联合也是提高NSCLC诊断效能的一个研究方向。外周血存在的主要问题有部分样本DNA含量较低（可望通过提高检测技术的敏感性来克服）以及缺乏器官特异性。

痰液含有来自肺和下呼吸道的脱落细胞，具有一定的特异性，痰液的甲基化位点也有较多报道。一项研究^[31]^确定痰液中4个甲基化位点（*APC*、*p16*、*HS3ST2*和*RASSF1A*）是NSCLC早期检测的理想组合，AUC为0.8。吸烟人群是肺癌发病的危险人群，且这部分人痰液较多，不需要诱导即可获得，因此比较适合早期筛查。Belinsky等^[32]^报道，肺癌患者痰液中检测到*MGMT*、*RASSF1A*、*DAPK*和*PAX5α*中三者及以上的位点甲基化，比非肿瘤吸烟者痰液的几率高6.2倍。进一步的前瞻性研究表明，在肺癌诊断前18个月收集的痰液样本中，检测到*p16*、*MGMT*、*DAPK*、*RASSF1A*、*PAX5β*和*GATA5*这6个基因中三者及以上的位点甲基化，肺癌风险增加6.5倍^[33]^。但是痰液主要来自于中央的肺门部位，故可能不适于检测腺癌，因后者常发生于肺的边缘部位。

支气管灌洗液（bronchoalveolar lavage, BAL）是另一个可供选择的研究样本，由于支气管镜是所有可疑肺癌患者必做的检查，因此，BAL也较容易获得，并可能部分含有肺特异性的肺癌细胞或DNA。Kim等^[34]^对85例NSCLC的BAL研究表明，68%的样本至少检出*p16*、*RARβ*、*H-cadherin*和*RASSF1A*其中之一的甲基化；另一项研究^[35]^报道，247例可疑肺癌患者（确诊89例）的BAL中，联合*APC*、*p16*和*RASSF1*甲基化检测的诊断敏感性为53%，特异性为99%。

## 4 DNA甲基化与NSCLC预后及复发

多项研究表明，DNA甲基化与NSCLC临床分期或淋巴结转移有关，提示表观遗传学异常还参与肿瘤演进，可作为预后判断的指标。Tang等^[36]^报道，44%（59/135）的Ⅰ期NSCLC组织表现出*DAPK*高甲基化，且5年生存率显著低于未甲基化的患者（0.46 *vs* 0.68;*P*=0.007）。Burbee等^[37]^报道，30%（32/107）的NSCLC手术标本检出*RASSF1A*甲基化，且与患者预后不良有关。Seng等^[38]^在NSCLC中研究位于染色体3p的5个抑癌基因（*RASSF1A*、*BLU*、*RARβ*、*DLEC1*和*hMLH1*）的甲基化，发现*DLEC1*和*hMLH1*的甲基化比例分别为38.7%和35.7%，二者均与肿瘤分期、淋巴结转移有关，是不良预后的独立影响因素。另外一项研究^[39]^发现*RXRG*基因甲基化在吸烟的NSCLC患者中，与预后不良相关，但在吸烟患者中，反而是保护因素。此外，NSCLC中报道较多的预后相关的甲基化位点还有*APC*、*ASC/TMS1*、*CDH1*、*CDH13*、*DAL-1*、*FHIT*、*MGMT*、*p16*、*RUNX3*、*CADM1/TSLC1*等^[40]^。值得注意的是全基因组的低甲基化与启动子区域的局部高甲基化一样，也可作为潜在的预后指标，最近的一项研究^[41]^在379例NSCLC组织中分析*APC*、*CDH13*、*RASSF1A*和*LINE-1*（long interspersed nuclear element 1，可代表全基因组甲基化水平）甲基化状态，发现*LINE-1*低甲基化是Ⅰa期NSCLC预后不良的独立因素。

甲基化与NSCLC复发的关系也有报道，Brock等^[42]^对术后40个月内复发的51例和无复发的116例Ⅰ期NSCLC标本，研究*p16*、*MGMT*、*DAPK*、*RASSF1A*、*CDH13*、*APC*和*ASC*的甲基化状态，结果发现，*p16*、*CDH13*、*RASSF1A*和*APC*在肿瘤组织或相应淋巴结（组织学正常）中的甲基化与肿瘤复发有关，并且不受年龄、性别、种族、手术方式、肿瘤大小、组织学类型及吸烟状态的影响；如果*p16*和*CDH13*甲基化同时在肿瘤组织和纵隔淋巴结检测到，复发的优势比（odds ratio, OR）是15.5。作者认为这些组织学正常的淋巴结中检测到DNA甲基化提示肿瘤微转移。

## 5 DNA甲基化与化疗敏感性

近来有研究^[43]^表明，个别基因的甲基化状态与治疗反应性有关，如甲基化对DNA修复基因*MGMT*和*hMLH*的表观遗传学失活，分别与烷化剂和5-FU的敏感性改变有关，对指导胶质瘤和大肠癌的治疗具有一定意义；有丝分裂检验点（checkpoint）控制基因*CHFR*的甲基化与微管抑制剂（如VP16）的敏感性有关。因此，可望通过甲基化标记确定不同的肿瘤亚型以利于个体化治疗。

*14-3-3σ*基因是主要的G_2_/M检验点控制基因，Ramírez等^[44]^在115例经顺铂联合吉西他滨治疗的进展期NSCLC患者中，研究血清DNA（化疗前收集）的*14-3-3σ*甲基化，结果表明，39例（34%）患者检出高甲基化，甲基化组中位生存期（15.1个月*vs* 9.8个月，*P*=0.004）和中位进展时间（8个月*vs* 6.3个月，*P*=0.027）更好，*Cox*回归模型证实，只有*14-3-3σ*甲基化状态和ECOG评分（PS）是影响预后的独立因素。*14-3-3σ*甲基化可能成为NSCLC铂类联合化疗的潜在预测指标。另一项类似研究^[45]^在吉西他滨（或联合其它药物）一线治疗的92例Ⅲb期和Ⅳ期NSCLC患者血清中，检测*APC1A*、*DAPK*、*FHIT*、*p14^ARF^*、*p16^INK4a^*、*RARβ*和*RASSF1A*的甲基化，尽管没有一个基因的甲基化与总生存期（overall survival, OS）有关，但在部分缓解（partial response, PR）的病例中，甲基化指数（methylation index, MI）>0.3的患者生存期更长[中位OS，（36.0±10.3）个月*vs*（17.4±4.3）个月，HR=0.12，95%CI：0.053-0.54）]，而在稳定和进展的病例中，二者无差别。提示一个或多个基因的表观遗传学异常可能在取得临床缓解的患者中发挥作用。并且，单一的*RASSF1A*基因甲基化在这一亚组中，也较未甲基化的患者生存期更长[中位OS，（33.6±10.4）个月*vs*（12.9±4.7）个月，*P*=0.0045），多因素分析表明，*RASSF1A*甲基化可作为PR患者的独立预后指标。

de Caceres等^[46]^通过基因表达芯片分析，证实顺铂诱导耐受的NSCLC细胞株中存在*IGFBP-3*的高甲基化失活，而在顺铂敏感的亲本NSCLC细胞株中，siRNA沉默*IGFBP-3*表达导致细胞对顺铂的耐受；进而，分析36例Ⅰ/Ⅱ期NSCLC组织的*IGFBP-3*甲基化，其中19例顺铂治疗无效，17例顺铂敏感，结果发现，*IGFBP-3*高甲基化更常见于顺铂耐受组（14/19 *vs* 2/17, *P* < 0.001）；并且，在Ⅰ期病例中，未甲基化的无疾病生存期（disease free survival, DFS）有增加的趋势。

## 6 DNA甲基化与NSCLC治疗

与基因突变等遗传学改变不同，DNA甲基化是可以逆转的过程。因此，在理论上，对癌前病变或肿瘤进行去甲基化处理可以恢复某些关键性抑癌基因的功能，从而起到预防和治疗肿瘤的作用。目前，表观遗传学的治疗已经在血液系统肿瘤中取得重大进展，如DNMT抑制剂decitabine（地西他滨）和HDAC抑制剂vorinostat分别被FDA批准用于骨髓异常增生综合征（myelodysplastic syndrome, MDS）^[47]^和皮肤T淋巴细胞瘤（cutaneous T-cell lymphoma）^[48]^的治疗。但在多种实体肿瘤中的结果并不令人满意，原因可能是对这些药物作用的机制了解不够，对适宜患者的选择以及最佳剂量等仍需要进一步探索。在肺癌中的相关研究也刚刚起步，一项由美国国立癌症研究所支持的I/Ⅱ期临床试验^[49]^正在Johns Hopkins大学进行，初步结果显示，Aza-dC联合HDAC抑制剂entinostat使部分难治的进展期NSCLC患者获益，并且耐受性较好。

## 7 DNA甲基化与microRNA

microRNA（miRNA）是一类长度为20个-24个核苷酸的非编码小分子RNA，主要通过与靶基因3’端非编码区发生不完全配对，从而降解靶基因mRNA或抑制其翻译，参与调控个体发育、细胞凋亡、细胞增殖和分化等生命活动^[50]^。miRNA的表达失调导致癌基因或抑癌基因异常活化和抑制是肿瘤发生发展中的常见事件。研究表明DNA甲基化也是miRNA表达失活的机制之一。在肺癌中报道的两个位点是miR-34a和miR124a。miR-34家族（miR-34a、miR-34b和miR-34c）是构成p53信号网络的一部分，miR-34a启动子区CpG岛甲基化在29.1%（7/24）的肺癌细胞株中检测到^[51]^；Gallardo等^[52]^在70例术后NSCLC标本中检测miR-34家族的表达和甲基化状态，发现高甲基化引起的miR-34a低表达（*P*=0.008）与肿瘤复发有关（*P*=0.04）。miR124a是CDK6（cyclin D kinase 6）的调控因子，*miR124a*甲基化在4/6的肺癌细胞系和13/27（48%）的原发肿瘤中检测到，而正常肺组织和细胞株均未甲基化，并且，*miR124a*高甲基化与转录失活有关，5-Aza-dC处理使其表达恢复^[53]^。

另一方面，Fabbri等^[54]^发现，miRNA不仅仅是甲基化调控的靶点，反过来也参与对DNA甲基化的调控。DNMT3A和DNMT3B是miR-29s（miR-29a, -29b, -29c）的调控靶点，NSCLC细胞（A549和H1299）中miR-29s过表达导致DNMT3A和-3B表达下降，且NSCLC组织中DNMT3A和DNMT3B mRNA表达与miR-29s水平呈负相关。转染miR-29a、miR-29b和miR-29c到A549细胞，均导致全基因组甲基化水平的降低，与Dnmt1抑制剂decitabine的效应类似，同时，部分抑癌基因，如FHIT和WWOX启动子区高甲基化得以逆转，表达恢复。

## 8 结语

随着人类进入功能基因组时代，在时间和空间上调控基因表达的表观遗传学研究受到了广泛重视。DNA甲基化作为表观遗传修饰的主要方式，在肺癌等肿瘤疾病的研究中也有了长足的进展，为肿瘤早期诊断、预后判断和干预治疗提供了新的思路，展现了良好的前景。但仍有许多问题亟待解决，例如，作为肿瘤标记物，目前报道的高频特异性位点极少，NSCLC中鉴定的大量甲基化位点还需要临床验证；甲基化检测方法众多，但各自都有一些缺点和局限性，难以普及于临床；另外，DNMT抑制剂缺乏特异性，可能导致原本处于抑制状态的一些基因恢复活性，促进癌变的发生，具有潜在的副作用。因此，需要继续进行DNA甲基化在NSCLC中的深入研究，揭示甲基化在肿瘤发生发展中的作用机制，使其可望在将来成为NSCLC诊断及治疗的有效工具。
